# Contribution of tissue transglutaminase to the severity of hepatic fibrosis resulting from *Schistosoma japonicum* infection through the regulation of IL-33/ST2 expression

**DOI:** 10.1186/s13071-019-3542-4

**Published:** 2019-06-14

**Authors:** Zhi-Yong Li, LinZhuo Xiao, GuiYing Lin, JuanJuan Tang, YuQiang Chen, Lan Chen, BaoQi Li, MeiLing Wu, ShuYan Liu, ChuQin Huang, Dominique Ferrandon, Zi Li

**Affiliations:** 1 Sino-French Hoffmann Institute, Guangzhou Medical University, Guangzhou, 511436 Guangdong Province People’s Republic of China; 2 RIDI UPR9022 du CNRS, Université de Strasbourg, 67000 Strasbourg, France

**Keywords:** Tissue transglutaminase, IL-33, ST2, Liver fibrosis, *Schistosoma japonicum*

## Abstract

**Background:**

Tissue transglutaminase (tTG)-regulating IL-13 plays an important role in the pathogenesis of liver fibrosis resulting from *Schistosoma japonicum* (*Sj*) infection. IL-33 and its receptor ST2 are involved in Th2-biased immune responses through the release of IL-5 and IL-13 and subsequent hepatic granuloma pathology induced by *Sj* infection. However, the relationship between tTG, IL-33/ST2, and liver fibrosis during *Schistosoma* infection has not been established.

**Results:**

This study investigated the link between tTG and IL-33/ST2 in the induction of liver fibrogenesis during *Sj* infection in mice. The extent of liver fibrosis coincided with an increase in tTG and IL-33/ST2 expression in the liver of infected mice between five to eight weeks, with a peak of correlation at six weeks after *Sj* infection. The inhibition of tTG activity through cystamine administration or gene knockout alleviated the level of TLR4, NF-κB pathway molecules, IL-33/ST2, and the severity of liver fibrosis resulting from *Sj* infection.

**Conclusions:**

These results indicate that during *Sj* infection tTG may control liver fibrosis at least partially through TLR4, NF-κB pathway activation and then IL-33/ST2. tTG, IL-33 or ST2 might be promising drug targets against liver fibrosis induced by *Sj* infection.

## Background

Schistosomiasis is a parasitic disease affecting more than 200 million people worldwide according to a report of the World Health Organization in 2016 [1]. The main pathogenic species causing schistosomiasis are *Schistosoma japonicum* (*Sj*), *S. mansoni* (*Sm*) and *S. haematobium* (*Sh*). In China, *Sj* is the only parasite causing this disease. Infection of *Sj* leads to severe liver granulomatous immunopathology and fibrosis induced by eggs trapped in the liver [2], which are the primary cause of morbidity and mortality. Th1 and Th2 responses play key roles in the immunity to infections. A robust Th2 response to *Schistosoma* egg antigens is a major factor leading to liver fibrosis, while the Th1-type response remains moderate [3]. Interleukin-33 (IL-33), a member of the IL-1 superfamily [4, 5], can activate mast cells, Th2, group 2 innate lymphoid cells (ILC2) and eosinophils and leads to the release of IL-5 and IL-13, the major cytokines mediating the Th2-biased immune response [6–10]. The hepatocyte is a major source of IL-33 in mice liver fibrosis models induced by thioacetamide or carbon tetrachloride treatment [11]. Suppression of tumorigenicity 2 (ST2) is a high affinity receptor family member for IL-33 [12]. Recently, it has been shown that IL-33 and ST2 are involved in Th2-biased immune responses by triggering on the one hand IL-5 and IL-13 release and on the other hand hepatic granuloma pathology induced by *Sj* infection [13, 14]. However, the actual mechanism used by IL-33 to promote the formation of granuloma and fibrosis in the liver during *Sj* infection needs to be further delineated.

Tissue transglutaminase (tTG) is a distinctive member of the transglutaminase family, as it is ubiquitously expressed, catalyzes a number of different reactions and is involved in mediating numerous molecular processes, including cell death, signaling, cytoskeleton rearrangements, ECM stabilization, and fibrosis [15, 16]. We recently reported that tTG-induced regulation of IL-13 plays an important role in the pathogenesis of liver fibrosis resulting from *Sj* infection [17, 18].

Moreover, tTG reportedly induces IL-33 expression and a subsequent Th2 response in the allergen response of airway epithelial cells [19]. Given that both IL-33/ST2 and tTG have been implicated in fibrosis through the release of IL-13, this study was undertaken to genetically validate the role of tTG in liver fibrosis during *Sj* infection and the relationship between tTG and IL-33/ST2. Herein, we observed that the extent of liver fibrosis in *Sj* infection mice is consistent with the expression levels of tTG and IL-33/ST2. Importantly, we further demonstrated that tTG activity inhibition or tTG knockout leads to a decreased expression level of IL-33/ST2 and alleviated severity of liver fibrosis. These findings indicate that tTG contributes to the severity of hepatic fibrosis resulting from *Sj* infection by regulating IL-33 and ST2 expression.

## Methods

### Reagents

Sirius red staining (connective tissue staining) kit was purchased from Abcam (ab150681, Cambridge, UK). Trizol was obtained from Life Technologies (Waltham, USA). SYBR® Premix Ex Taq™ II (RR820A) and PrimeScript™ RT reagent Kit with gDNA Eraser (RR047A) were from TaKaRa Biotechnology Co. Ltd. (Dalian, China). The antibodies used were as follows: anti-tTG (ab109200, Abcam), anti-IL-33(ab54385, Abcam), Anti-ST2 (sc-74296, Santa Cruz Biotechnology, Dallas, USA), anti-COL I (14695-I-AP, Abcam, USA), anti-TLR4 (Ab47093, Abcam, USA), anti-p-p65 (sc136548, Santa Cruz Biotechnology), anti-p-65 (sc8008, Santa Cruz Biotechnology), anti-p-IKKα/β (ab178870, Abcam), anti-IKKγ (SC166700, Santa Cruz Biotechnology), anti-GAPDH (ab181603, Abcam), and HRP-conjugated secondary antibodies of mice or rabbit IgG (35552 and 35510, Invitrogen, Waltham, USA). Bicinchoninic acid (BCA) Protein Assay Kit was purchased from Guangzhou Dingguo Biotechnology (Guangzhou, China). Polyvinylidene fluoride membrane (PVDF) (ISEQ00010) was purchased from Merck Millipore (Darmstadt, Germany). 3,3′-diaminobenzidine (DAB) substrate kit was purchased from Gene Tech Company Limited (Shanghai, China). Cystamine (CTM), the inhibitor of tTG enzyme activity, TAK242, a TLR4 inhibitor were purchased from Sigma-Aldrich (St. Louis, USA) and Shanghai Haoyuan Chemoexpress (Shanghai, China), respectively.

### Mice, parasite infection and CTM, TAK242 treatment

Six to eight-week-old female C57BL/6 wild-type mice were fed with commercially available diet and housed in a controlled environment at 25 ± 2 °C. They were infected per-cutaneously through the abdomen with 20 ± 3 *Sj* cercariae of the Chinese mainland strain. The mice developed liver granuloma, then acute and advanced fibrosis at week 5, 6 and 8 post-infection (n = 6–10), respectively. The uninfected mice were used as normal controls (*n* = 6). According to our previous experiments, inhibition of tTG with CTM at the early stage of *Sj* infection strikingly decreases the extent of liver fibrosis [17, 20]. We treated mice with CTM (10^−2^ M, 100 µl per mouse, *n* = 4) in PBS by intraperitoneal injection once a day from day 3 to day 10, while the infection control group (*n* = 10) only received PBS. Two non-infected control mice groups were treated with CTM (*n* = 5) and PBS (*n* = 6) respectively. TAK242 treatment and the allocation of mice to each group were performed according to our previous study [20]. All mice were sacrificed at indicated time points and liver tissues were collected for further analysis.

### Measurement of collagen deposition area in mice liver *via* Sirius red staining

Fresh hepatic tissues were fixed in 4% paraformaldehyde for 24 h, and then embedded with paraffin. The 4 µm liver sections were prepared and stained with Sirius red to evaluate the extent of collagen deposition, which mirrors the severity of liver fibrosis. The morphometric collagen area of Sj single egg granuloma as displayed in deep red was measured and analyzed by Image J software. Each stained section was evaluated in double-blind fashion by two independent researchers.

### RNA isolation and quantitative PCR

Total RNA was extracted from fresh liver of mice using Trizol reagent. Genomic DNA was removed, and then cDNA was synthesized using PrimeScript™ RT reagent Kit with gDNA Eraser. The relative RNA expression level of target genes was measured by real-time quantitative reverse transcription polymerase chain reaction (q-PCR) with SYBR® Premix Ex Taq™ II kit and the CFX96™ real-time system (BIO-RAD, San Francisco, USA) according to the manufacturer’s procedure. The following primer sequences were used in quantitative-RT PCR: mouse-GAPDH (GenBank: M32599, forward: 5′-TGT GTC CGT CGT GGA TCT GA-3′; reverse: 5′-AAC CTC AAT CCA GAA CAC TT-3′); mouse-tTG (GenBank: NM-009373.3, forward: 5′-CTG AAC AAA CTG GCA GAG AAA G-3′; reverse: 5′-CAG AGC AGG AGA CGA CAC T-3′); mouse-IL-33(GenBank: NM-001360725.1, forward: 5′-CCT CCC TGA GTA CAT ACA ATG ACC-3′; reverse: 5′-GTA GTA GCA CCT GGT CTT GCT CTT-3′); mouse-ST2 (GenBank: NM001294171.1, forward: 5′-ATC ACC GAA GCA TCT ACT AC-3′; reverse: 5′-AAC CTC AAT CCA GAA CAC TT-3′). The RNA expression level of individual genes was normalized to GAPDH and analyzed using the 2^−ΔΔCt^ data analysis method.

### Western blotting

Fresh liver tissues of mice were placed on ice for grinding and crushing, and the total protein homogenate was prepared and then treated with protein extraction solution (RIPA:PMSF = 100:1). In order to remove nucleic acid, the protein homogenate was placed on ice for 1 h, and then centrifuged at high speed at 4 °C for 15 min. Protein concentrations were determined using the BCA Protein Assay Kit. Equal amounts of total proteins were resolved by 10% SDS-PAGE, and next transferred to PVDF. After blocking with 5% milk or bovine serum albumin (BSA), and incubating with the primary and secondary antibodies, the target protein bands were revealed by enhanced chemiluminescence (ECL) reagent. The primary antibodies used were as follows: anti-GAPDH, -tTG, -IL-33, -ST2 and anti-Col I.

### Immunohistochemical (IHC) assay with semi-quantitative analysis

Endogenous peroxidase in mouse liver sections was blocked with 3% hydrogen peroxide (H_2_O_2_). Immunohistochemical (IHC) staining assay was used to determine the expression level and location of IL-33 and ST2 in the mice liver tissue using anti-IL-33 (1:500) and anti-ST2 (1:500) primary antibodies, followed by HRP-conjugated secondary anti-rabbit or anti-mouse antibodies. The images were observed and captured with an optical microscope equipped with a camera (Olympus, Tokyo, Japan). The semi-quantitative analysis was determined by image J software and modified H-score [20].

### Measurement and analysis of *Sj* egg load

Liver tissue sections were prepared and stained with Sirius red. The average total number of living eggs (with miracidium inside) in five fields (at a magnification of 200×) of each section was counted. Average number of eggs of each liver section was calculated and reported as the *Sj* egg load.

### Statistical analysis

The results are given as the mean ± standard error of the mean (SEM) or standard deviation (SD) as noted of several replicates or experiments. SPSS22.0 software was used to analyze the statistical differences among multiple groups and the Studentʼs t-test, Mann-Whitney U-test were used to compare pairs of experiments. A *P-*value of < 0.05 was considered statistically significant.

## Results

### The level of tTG and IL-33/ST2 is consistent with the severity of hepatic fibrosis resulting from *Sj* infection

**Fig. 1:**
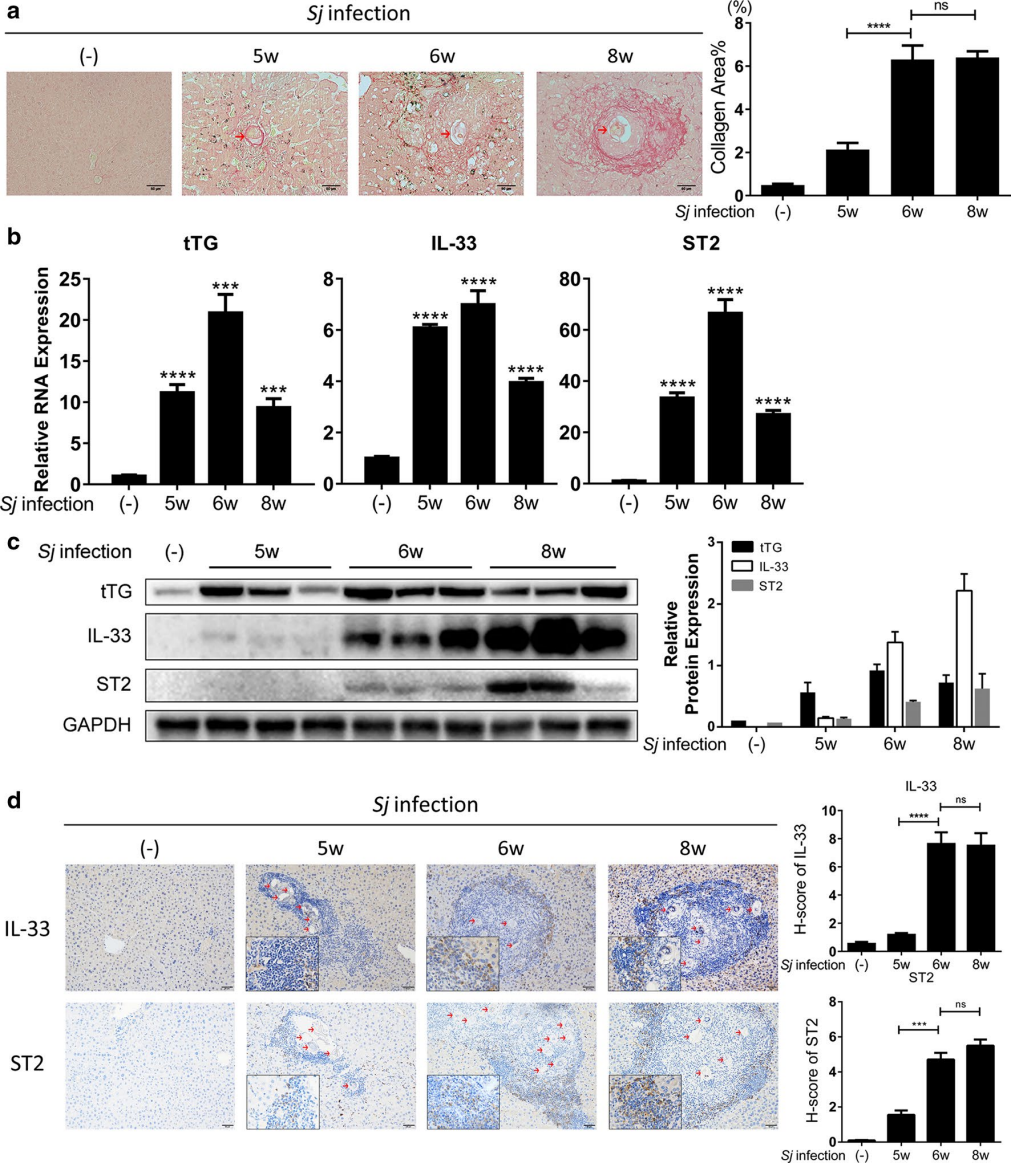
The level of tTG and IL-33/ST2 is consistent with the severity of hepatic fibrosis post *Sj* infection.*C57BL/6* mice were infected with 20 ± 3 cercariae of *Sj* for 0, 5, 6 and 8 weeks. Hepatic tissue sections were fixed and stained with Sirius red, or anti-IL-33 and anti-ST2 primary antibody. (**a**, *left*) A typical Sirius red staining (200×, *scale-bar*: 50 μm) of *Sj* single egg granuloma is shown. (**a**, *right*) Percentage of morphometric collagen areas around *Sj* single egg (stained with positive red) was calculated and shown (t-test, ****P < 0.0001). **b** The relative RNA expression levels of tTG, IL-33 and ST2 in liver tissue of mice infected with *Sj* at indicated time courses were measured by RT-qPCR. *GAPDH* expression levels were taken for reference (t-test, ***P = 0.0001, ****P < 0.0001). **c** The protein expression level of tTG, IL-33 and ST2 in mice liver homogenates detected by Western blotting are displayed on the left panel, and semi-quantitative analysis is shown on the right panel. GAPDH was used as a loading control. **d** The typical protein expression levels and location of IL-33 and ST2 in mice liver tissue were determined by immunohistochemical (IHC) assay at weeks 5, 6 and 8 after *Sj* infection were shown (200× and 400×, left, *scale-bar*: 50 μm), and the semi-quantitative analysis of these proteins was analyzed using a modified H score (right) [t-test; ****P < 0.0001, ***P = 0.0004, ns, no significance, *P* = 0.9228 (upper panel), *P* = 0.1711 (lower panel)]. Data are presented as the mean ± SEM from 3–6 mice per group. All *in vitro* experiments were performed twice or thrice. Eggs are indicated by red arrows

The severity of liver granuloma and fibrosis around *Sj* single egg was evaluated by measuring collagen area using Sirius red staining (in red, shown in Fig. 1a, left panel). At week (wk) 6 and 8 of *Sj* infection, the extent of hepatic fibrosis reached significantly high levels (t-test: wk 6 and wk 8 *vs* wk 5: *t*_(116)_ = 6.233, *P* < 0.0001) (Fig. 1a), in agreement with our previous studies [17, 20]. However, there were no significant differences between week 6 and week 8 (t-test: wk 8 *vs* wk 6: *t*_(195)_ = 0.1558, *P* = 0.8763).

To evaluate the correlation between tTG, IL-33/ST2 expression and hepatic fibrosis, the expression level of tTG, IL-33 and ST2 in mice liver were measured using RT-qPCR (Fig. 1b) [t-test: TG2 (wk 5 *vs* (−): *t*_(4)_ = 17.93, *P* < 0.0001; wk 6 *vs* (−): *t*_(4)_ = 15.12, *P* = 0.0001; wk 8 *vs* (−): *t*_(4)_ = 13.32, *P* = 0.0001); IL-33 (wk 5 *vs* (−): *t*_(4)_ = 57.8, *P* < 0.0001; wk 6 *vs* (−): *t*_(4)_ = 18.74, *P* < 0.0001; wk 8 *vs* (−): *t*_(4)_ = 28.27, *P* < 0.0001); ST2 (wk 5 *vs* (−): *t*_(4)_ = 28.29, *P* < 0.0001; wk 6 *vs* (−): *t*_(4)_ = 21.37, *P* < 0.0001; wk 8 *vs* (−): *t*_(4)_ = 28.14, *P* < 0.0001)], Western blotting (Fig. 1c) and IHC assay (Fig. 1d) in *Sj*-infected and uninfected mice groups. Compared with the uninfected mice, the RNA and protein expression levels of tTG, IL-33 and ST2 significantly increased in *Sj*-infected mice, and the expression level of the three molecules consistently peaked at 6 weeks of infection [t-test: IL-33 (wk 6 *vs* wk 5: *t*_(225)_ = 5.912, *P* < 0.0001; wk 8 *vs* wk 6: *t*_(239)_ = 0.09706, *P* = 0.9228); ST2 (wk 6 *vs* wk 5: *t*_(61)_ = 3.776, *P* = 0.0004; wk 8 *vs* wk 6: *t*_(162)_ = 1.375, *P* = 0.1711)].

IL-33, an alarmin, has been reported to be widely expressed in the cells of liver tissue, while ST2 expression is limited to ILC2 and macrophages [11, 14]. Herein, IL-33, ST2 expression was found mainly in the cells surrounding the *Sj* eggs. IL-33 was located in cells at the outer rim of granulomas while ST2 was found relatively more in cells inside.

### tTG is required for the development of liver fibrosis and IL-33/ST2 full induction during *Sj* infection

The role of tTG in hepatic fibrosis has been reported in several studies [15–17, 20]; however, genetic evidence of the involvement of this enzyme in *Sj*-induced liver fibrosis is lacking and its specific regulatory mechanisms remain incompletely understood. IL-33 and ST2 have been reported to be associated with liver fibrosis [11]. We tested whether tTG regulates IL-33 and ST2 expression during liver fibrosis induced by *Sj* infection either using tTG knock-out mice or by inhibiting tTGʼs enzymatic activity.

**Fig. 2:**
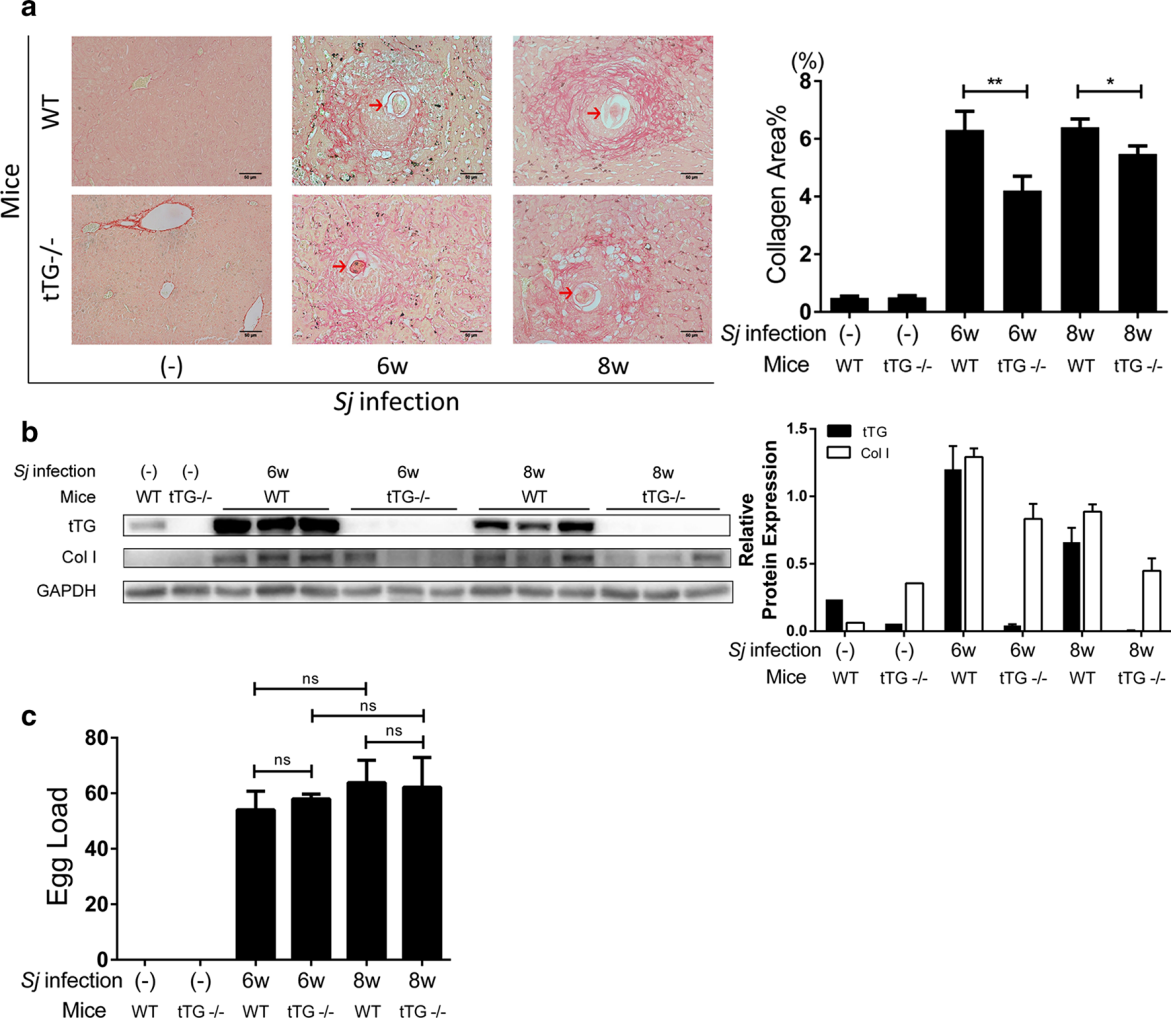
tTG deletion alleviates the severity of *Sj*-induced hepatic fibrosis. Wild-type (WT) and tTG deficient (tTG-/-) mice were infected with 20 ± 3 cercariae of *Sj* for 0, 6 and 8 weeks. **a** Typical *Sj* single egg granuloma stained by Sirius red (200×, *scale-bar*: 50 μm) is shown on the left panel. The percentage of morphometric collagen areas around *Sj* single egg was calculated and is shown on the right panel (Mann-Whitney U-test, **P = 0.001, *P = 0.0492). **b** The protein expression levels of tTG and Col1 in mice liver homogenates detected by Western blotting are displayed on the left panel, and semi-quantitative analysis is shown on the right panel. GAPDH was used as loading control. **c** The average *Sj* egg numbers present in each liver section is shown. Data are presented as the mean ± SD from approximately three to six mice per group. All *in vitro* experiments were performed twice or thrice. Red arrows indicate the *Sj* eggs (t-test, ns)

Compared with wild type (WT) mice, the extent of liver fibrosis in tTG null mice after *Sj* infection was decreased significantly (Mann-Whitney U-test: WT wk 6 *vs* tTG-/- wk 6: *U*_(139)_ = 1500.05, *Z* = -3.249, *P* = 0.0010; WT wk 8 *vs* tTG-/- wk 8: *U*_(257)_ = 6941.5, *Z* = -1.968, *P* = 0.0492) (Fig. 2a). Western blotting revealed that the expression level of Collagen I in the liver of tTG null mice after *Sj* infection decreased (Fig. 2b), in agreement with the Sirius Red staining analysis. The *Sj* egg load showed no significant differences (t-test: WT wk 6 *vs* tTG-/- wk 6: *t*_(11)_ = 0.526, *P* = 0.6093; WT wk 8 *vs* tTG-/- wk 8: *t*_(12)_ = 0.117, *P* = 0.9088. WT wk 6 vs WT wk 8: *t*_(11)_ = 0.9406, *P* = 0.3671; tTG-/- wk 6 *vs* tTG-/- wk 8: *t*_(12)_ = 0.3315, *P* = 0.7460) in all *Sj* infected groups, whether expressing *tTG* or not (Fig. 2c). Thus, it can be ruled out that the lower extent of liver fibrosis in TG2 mutant mice was due to a lower number of *Sj* eggs being deposited in the liver

**Fig. 3:**
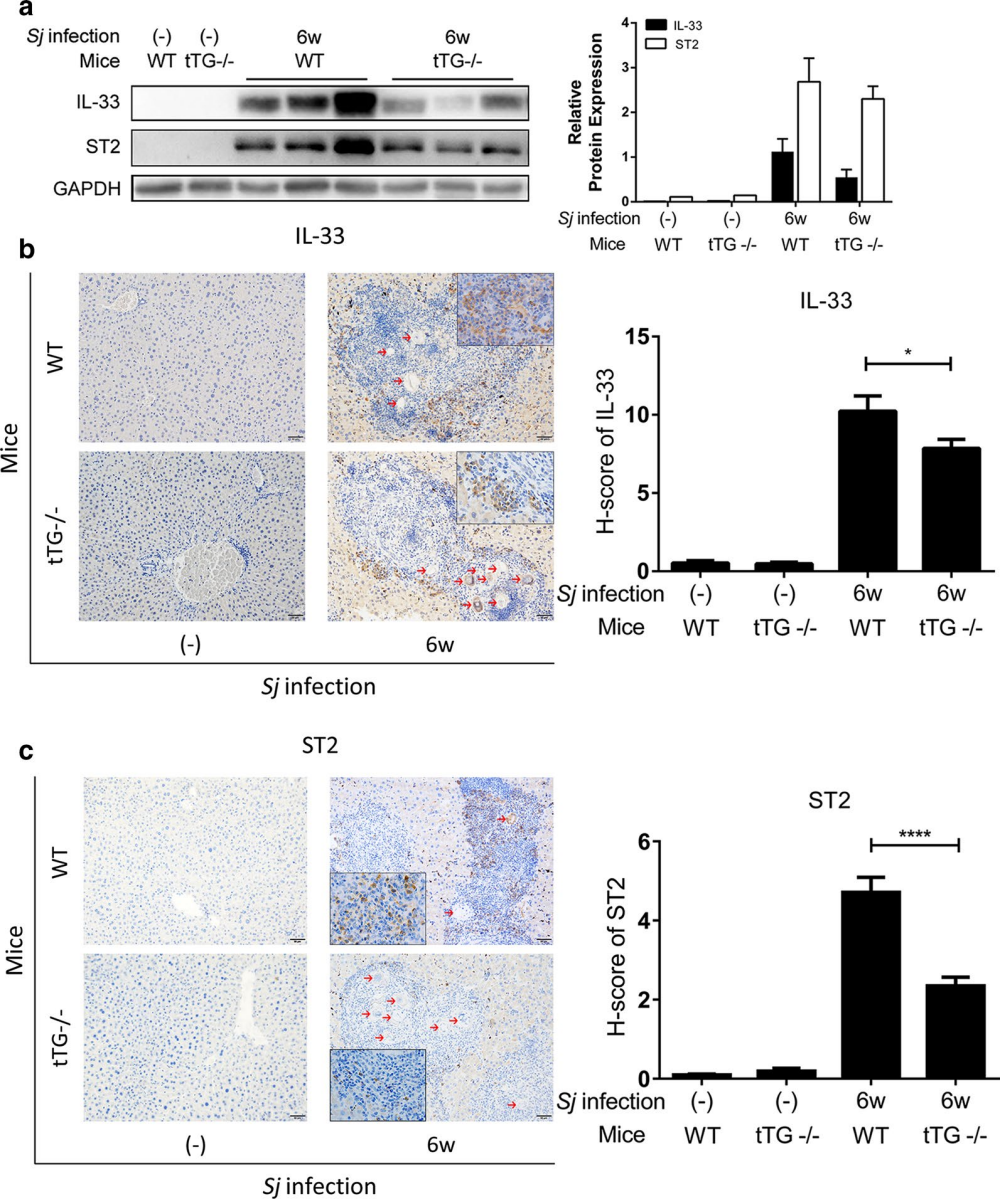
tTG knockout down-regulated the expression level of IL-33/ST2. **a** The protein level of IL-33 and ST2 in mice liver homogenates detected by Western blotting is shown on the left panel, and semi-quantitative analysis is displayed on the right panel. GAPDH was used as a loading control. **b**, **c** The protein expression levels and localization of IL-33 (**b**) and ST2 (**c**) in mice liver tissue were determined by IHC assay (200× and 400× (insets); *scale-bar*: 50 μm), and the quantification of the signal using modified H score analysis is shown on the right. Data are presented as the mean ± SEM from 3–6 mice per group. All *in vitro* experiments were performed three times. Red arrows indicate the *Sj* eggs (t-test, *P = 0.0360, ****P < 0.0001)

Compared to WT mice, IL-33 and ST2 protein expression level in livers of tTG-knockout mice was decreased significantly at 6-week *Sj* infection as monitored through Western blotting (Fig. 3a) and IHC assay [t-test: IL-33 (WT wk 6 *vs* tTG-/- wk 6: *t*_(147)_ = 2.117, *P* = 0.0360); ST2 (WT wk 6 *vs* tTG-/- wk 6: *t*_(78)_ = 4.361, *P* < 0.0001) (Fig. 3b, c). IL-33- and ST2-positive staining cells were mostly located on the outside rim and inside of granulomas, respectively.

### tTG activity, TLR4 and NF-κB pathway activation are involved in tTG-regulated IL-33/ST2 expression

**Fig. 4:**
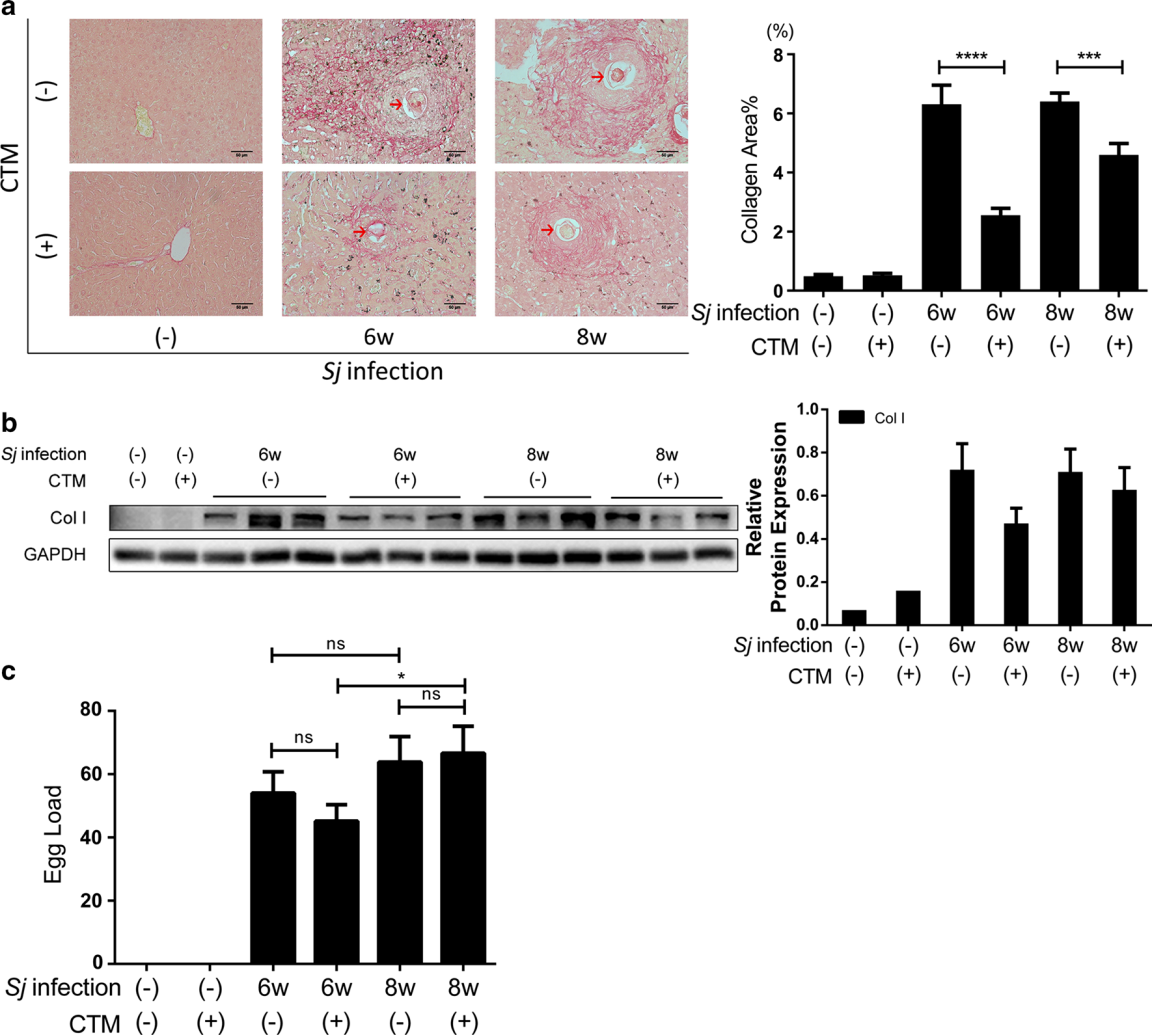
Suppression of tTG activity by cystamine treatment diminishes the extent of hepatic fibrosis. tTG activity in mice was inhibited by cystamine (CTM) through daily intraperitoneal injection from day 3 to day 10 after *Sj* infection. Mice were sacrificed at week 6. Non-infected mice with or without CTM treatment served as controls. **a** Representative Sirius red staining (200×, *scale-bar*: 50 μm) of *Sj* single egg granuloma is shown on the left panel. The percentage of morphometric collagen areas of single *Sj* egg granuloma is displayed on the right panel (t-test, ****P < 0.0001, ***P = 0.0003). **b** The protein expression level of Col1 in mice liver homogenates detected by Western blotting is displayed on the left panel, and the result of semi-quantitative analysis is shown on the right panel. GAPDH was used as loading control. **c** The average *Sj* egg number present in each liver section is shown. Data are presented as the mean ± SD. All experiments were performed twice or thrice. Red arrows indicate the *Sj* eggs (t-test, *P = 0.0496)

Cystamine (CTM), an inhibitor of tTG, selectively inhibits the activity of tTG by modifying a cysteine with disulfide interchange at the active site of tTG. To verify the effect of tTG enzyme deficiency on hepatic fibrosis in *C57BL/6* mice, we treated *Sj*-infected mice with CTM. Compared to mock-treated mice, the severity of hepatic fibrosis in *Sj*-infected mice for 6 and 8 weeks with CTM treatment was obviously alleviated as shown by Sirius red staining (Fig. 4a) and ColI Western blotting analysis (Fig. 4b) (t-test: WT wk 6 *vs* CTM wk 6: *t*_(175)_ = 6.85, *P* < 0.0001; WT wk 8 *vs* CTM wk 8: *t*_(213)_ = 3.661, *P* = 0.0003). As for the knock-out mice, CTM treatment did not change the *Sj* egg load (Fig. 4c) (t-test: WT wk 6 *vs* CTM wk 6: *t*_(15)_ = 1.054, *P* = 0.3085; WT wk 8 *vs* CTM wk 8: *t*_(8)_ = 0.2314, *P* = 0.8228; WT wk 6 *vs* WT wk 8: *t*_(11)_ = 0.9406, *P* = 0.3671; CTM wk 6 *vs* CTM wk 8: *t*_(12)_ = 2.184, *P* = 0.0496).

**Fig. 5:**
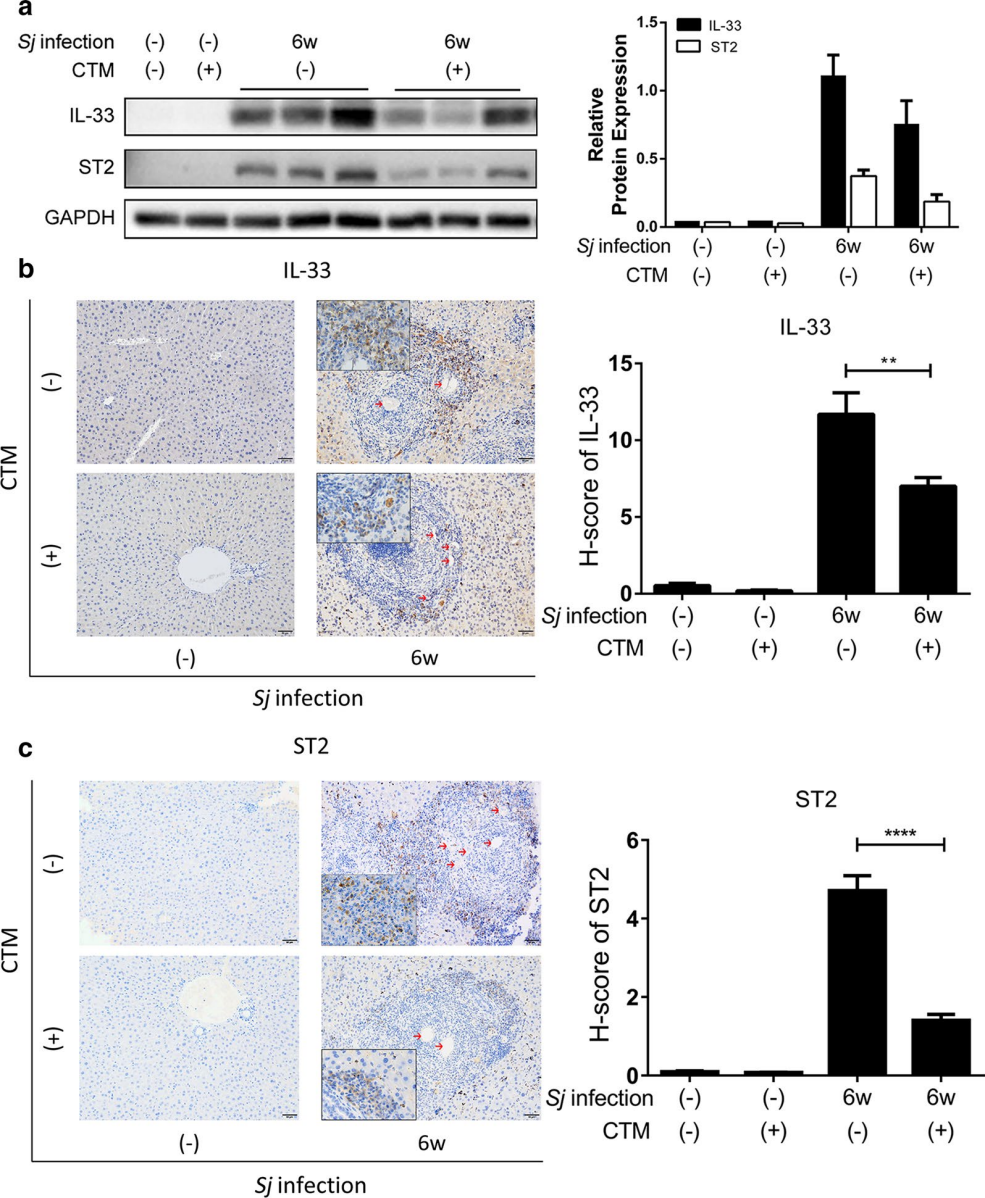
Suppression of tTG activity decreases the expression level of IL-33/ST2. **a** The protein expression levels of IL-33 and ST2 in mice liver homogenates detected by Western blotting are displayed on the left panel, and the result of semi-quantitative analysis is shown on the right panel. GAPDH was used as a loading control. **b**, **c** The protein expression levels and location of IL-33 (B) and ST2 (C) in mice liver tissue were determined by IHC assay (200× and 400× left, *scale-bar*: 50 μm), and the result of semi-quantitative analysis of the protein signal using modified H score is shown on the right panel. Data are presented as the mean ± SD. All experiments were performed twice or thrice. Red arrows indicate the *Sj* eggs (t-test, **P = 0.0014, ****P < 0.0001)

The protein expression levels of IL-33 and ST2 in *Sj*-infected mice liver with CTM treatment, were reduced as shown using Western blotting (Fig. 5a) and IHC assay [t-test: IL-33 (WT wk 6 *vs* CTM wk 6: *t*_(160)_ = 3.248, *P* = 0.0014); ST2 (WT wk 6 *vs* CTM wk 6: *t*_(134)_ = 9.354, *P* < 0.0001)] (Fig. 5b, c).

**Fig. 6:**
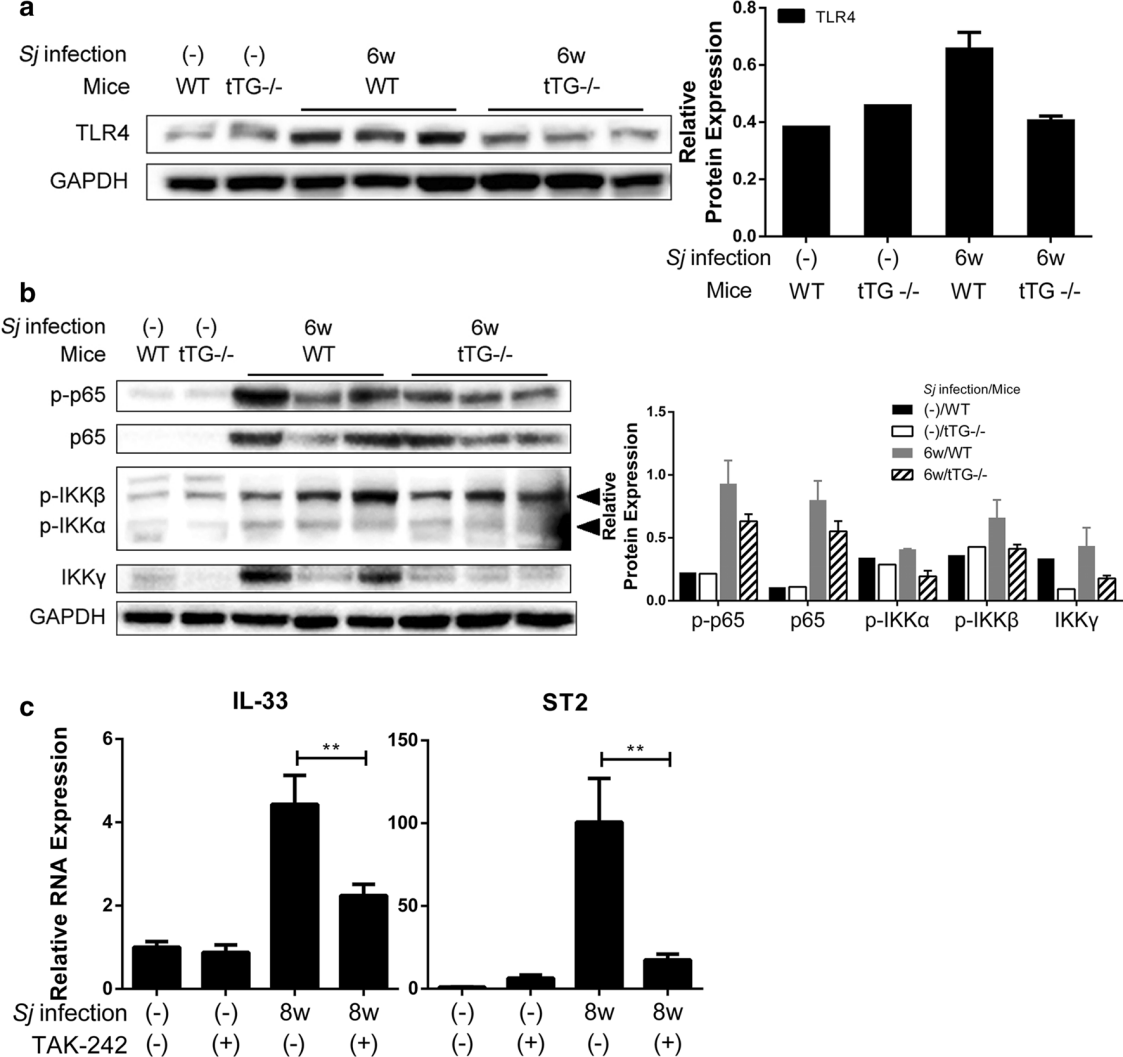
TLR4 and NF–κB pathway activation are involved in tTG-regulated IL-33/ST2 expression. **a** The protein expression level of TLR4 in indicated mice liver lysates detected by Western blotting is shown on the left panel, and the result of semi-quantitative analysis is displayed on the right panel. **b** The protein expression levels of p-p65, p-65, p-IKKα/β and IKKγ detected by Western blotting are shown on the left panel, and the result of semi-quantitative analysis is presented on the right panel. **c** The relative RNA expression levels of IL-33 and ST2 in liver tissue of mice infected with *Sj* for 6 weeks with or without exposure to the TLR4 inhibitor TAK242 were measured by RTqPCR. *Gapdh* was used as an internal reference [t-test, **P = 0.0069 (right panel); **P = 0.0057 (left panel)]

tTG has previously been reported to regulate TLR4 [20] and NF-κB pathway activation [16]. The protein expression levels of TLR4 (Fig. 6a), of some NF-κB pathway molecules including phosphorylated p65 (p-p65), p-IKKα, p-IKKβ, and IKKγ (Fig. 6b) were lowered in tTG-null mice compared to wild type (WT) mice after *Sj* infection. RNA expression levels of IL-33 and ST2 in mice liver induced by *Sj* infection were strongly reduced when TLR4 signaling was inhibited by TAK242 drug treatment [t-test: IL-33 (WT wk 8 *vs* TAK wk 8: *t*_(4)_ = 5.107, *P* = 0.0069); ST2 (WT wk 8 *vs* TAK wk 8: *t*_(4)_ = 5.406, *P* = 0.0057)] (Fig. 6c).

Taken together, these results suggest that tTG activity, TLR4, and NF-κB pathway activation are at least partially involved in regulating IL-33/ST2 induction.

## Discussion

In this study, we observed that tTG plays an important role in regulating IL-33/ST2 expression and alleviating the severity of liver fibrosis following *Sj* challenge. We rigorously established the role of tTG in the pathogenesis of liver fibrosis by using in parallel a genetic approach, tTG knockout mice, and a biochemical approach, the injection of cystamine, a selective inhibitor of tissue transglutaminase activity. Lowered IL-33 and ST2 expression following *Sj* infection was observed after implementing both strategies. Our results open the possibility that the fibrosis induced by *Sj* through the activation of tTG is mediated *via* the IL-33/ST2 axis.

In schistosomiasis, the immune response against *Schistosoma* eggs results in granuloma formation in liver and initiates the Th2-biased immunity that prevents acute mortality of the host but ultimately causes liver fibrosis [21]. IL-33 being a strong inducer of the Th2 immune response, the role of IL-33 in schistosomiasis has also been intensively studied. Mchedlidze et al. [11] reported that high levels of IL-33 in sera were critical for inducing the development of IL-13-dependent hepatic fibrosis in a murine model of *Sm* infection. Higher IL-33 expression also occurs in murine models of *Sj* [13, 14] and *Sh* infection [22]. Furthermore, a recent study showed that IL-33 needs to synergize with two other cytokine alarmins, thymic stromal lymphopoietin (TSLP) and IL-25, to induce IL-4/IL-13-dependent inflammation and fibrosis after *Sm* infection [23]. Our previous results showed the association of IL-13 and tTG for the development of liver fibrosis after *Sj* infection [17]. We did not use IL-13 or IL-33 knockout mice or neutralizing antibody to verify that IL-13 or IL-33 play a role in liver fibrosis during *Sj* infection as several publications have already reported that IL-13 [24] and IL-33 [11] mediate liver fibrosis during *Schistosoma* infection. Herein, the extent of liver fibrosis following *Sj* infection correlates with the expression level of IL-33 and ST2.

The roles of tTG in the pathogenesis of organ-specific and systemic inflammatory responses including fibrosis have been documented [25]. Although an important role for tTG in liver fibrosis has been suggested by several reports [15–17, 20, 25, 26], how tTG alleviates fibrosis is not thoroughly understood. In our previous studies, we have provided evidence that tTG regulates TGF-β1 of *Sj* origin and IL-13 of the host and documented the importance of tTG in liver fibrosis during *Sj* infection [17, 26]. The positive feedback regulation between tTG and Toll-Like Receptor 4 signaling in hepatic stellate cells correlates with liver fibrosis resulting from *Sj* infection [20]. In this study, our results utilizing tTG-/- mice and tTG inhibitor validated the importance of tTG during the pathogenesis of liver fibrosis induced by *Sj* infection and indicated that tTG is a putative novel disease target of parasite-induced liver fibrosis. tTG-null mice displayed reduced IL-33 expression following induction of allergic asthma compared to those in the WT control [19], which was also found in tTG-/- mice and tTG inhibitor-treated mice in our study of *Sj* parasitism. Our results taken in the light of this previous work suggests that tTG-IL-33/ST2 regulation may commonly happen in the pathogenesis of diseases displaying a Th2-biased immune response.

In mouse and human corneal epithelial cells, short ragweed pollen-stimulated IL-33 production was blocked by TLR4 antibody and a NF-κB inhibitor [27]. As shown in [27] and previous studies [16, 20], tTG is involved in TLR4 signaling [20] and NF-κB pathway activation [16]. Furthermore, the full induction of IL-33 and ST2 by *Sj* infection appears to be dependent on NF-κB signaling. How tTG regulates TLR4 or IL-33/ST2 will require further investigations, including the identification of the cell types involved in the process and the determination of whether the regulation is direct or indirect, given tTG’s multiple functions.

Herein, we evaluated collagen deposition area around *Sj* single egg but not multiple neighboring eggs included in one granuloma using Sirius red staining, which provides solid evidence of the extent of liver fibrosis. Here, the extent of liver fibrosis of *C57BL/6* mice between week 6 and 8 post-infection showed no significant difference, whereas it was not the case in *Sj*-infected *Balb/C* mice [17, 20, 26]. However, in these previous studies, we analyzed collagen area around single or multiple *Sj* eggs in a granuloma. Higher expression level of tTG at week 6 in comparison with week 5 and 8 post-infection in both *C57BL/6* and *Balb/C* [17] mice liver led us to evaluate its regulation relationship with IL-33/ST2 at week 6 post-infection. Our IHC results showed that IL-33 was mostly expressed in the cells of the outer rim of the granulomas, whereas ST2 was essentially expressed inside granulomas where macrophages are extensively present [28], in keeping with the report of Peng et al. [14] that ST2-expressing cells in the liver were mainly F4/80+ macrophages. As reported, IL-33 was critical for hepatic accumulation of ILC2 with high expression of ST2, and ILC2 mediated hepatic fibrosis resulting from *Sm* eggs challenge [11]. The role and mechanisms of ILC2 in liver fibrosis during *Sj* infection also deserve further study.

## Conclusions

In summary, we have validated the importance of tTG in liver fibrosis during *Sj* infection through knockout mice and inhibitor treatment. Our data suggest that tTG may function at least partially through the indirect induction of IL-33 and ST2 expression. The modulation of tTG activity with a consequent diminution of fibrosis in the liver may represent a potential therapeutic approach to hepatic granuloma pathology caused by *S. japonicum*, in as much as the absence of tTG does not appear to be required to control *Sj* egg numbers in infected livers.

## Data Availability

The datasets supporting the conclusions of this article are included within the article.

## Abbreviations

tTG: Tissue transglutaminase
ST2: suppression of tumorigenicity 2
*Sj*: *Schistosoma japonicum*
IL-33: Interleukin-33
CTM: Cystamine
TLR4: Toll like receptor 4
NF-κB: nuclear factor-kappa B
IKK: I-kappa-B-kinase
